# A qualitative study on operational challenges of Iranian affiliated mobile health clinics in Iraq during Arbaeen

**DOI:** 10.1038/s41598-025-22820-7

**Published:** 2025-11-06

**Authors:** Mohammad Amerzadeh, Seyed Saeed Tabatabaee

**Affiliations:** 1 Non-Communicable Diseases Research Center, Research Institute for Prevention of Non-Communicable Diseases, Qazvin University of Medical Sciences, Qazvin, Iran; 2 Social Determinants of Health Research Center, Mashhad University of Medical Sciences, Mashhad, Iran; 3 Department of Management Sciences and Health Economics, School of Health, Mashhad University of Medical Sciences, Mashhad, Iran

**Keywords:** Healthcare services, Challenges, Mass gatherings, Clinics, Health policy, Public health

## Abstract

Every year, between 17 and 20 million individuals participate in a religious pilgrimage called Arbaeen in Iraq. Providing healthcare services during this large gathering can pose various challenges. This study aimed to elucidate the challenges involved in delivering healthcare services in Iranian-affiliated mobile clinics based in Iraq during the Arbaeen ceremony from the Perspective of Healthcare Providers. In this qualitative study, 19 healthcare providers working in mobile clinics participated through purposive sampling. Data were collected through face-to-face interviews. Data collection continued until saturation. The content analysis method was employed for data analysis, using MAXQDA software (version 2020). The challenges faced by mobile healthcare service providers during the Arbaeen were categorized into three main categories: infrastructure, diseases and treatment and pilgrims. Within these categories, nine subcategories were identified: human resources, physical space, capital medical equipment, medications and medical supplies, communicable diseases, non-communicable diseases, inadequate understanding of potential hazards by pilgrims, physical disabilities of some pilgrims and medication-related issues. Planning and coordination at three levels: before, during, and after the Arbaeen, in the areas of infrastructure (human resources and physical resources), prevention and control of communicable and non-communicable diseases, procurement of drugs and medical supplies, and health education for pilgrims through various channels, as well as intra- and inter-sectorial coordination, can contribute to the effective improvement of service quality.

**Supplementary Information:**

The online version contains supplementary material available at 10.1038/s41598-025-22820-7.

## Introduction

The World Health Organization (WHO) defines mass gatherings as a specific number of individuals gathering at a particular location for a specific purpose within a defined and limited period. Communities involved in providing services and responding to mass gatherings require additional efforts, planning, and intensified actions^1^. Arbaeen is one of the largest human gatherings, held annually in Iraq with approximately 17 to 20 million participants. The participants of the Arbaeen walk from various cities in Iraq and neighboring countries to the city of Karbala, located in central Iraq^2^.

Every large human gathering can pose multiple public health challenges for participants, the host country, and the healthcare system^3^, and it can carry global health risks as well. Respiratory tract infections are the most common type of infectious diseases during the Hajj pilgrimage, affecting 50 to 93% of the participants^4^. In the large gathering of Touba, the largest mass gathering in West Africa, individuals who had visited healthcare facilities in Touba exhibited a prevalence of diseases with respiratory and gastrointestinal symptoms^5^. The situation of the public gathering during Arbaeen can be different from other large gatherings because much of the food and drinks distributed to participants on the streets or in temporary tents are prepared by volunteers, sometimes without observing hygiene principles, which leads to the spread of gastrointestinal infections during Arbaeen^2^. Additionally, the prevalence of cholera in Iraq in 2016 raises concerns about the further spread of these infections during Arbaeen^6^. In this type of ceremony, where participants from different nations, regions, and cultures come together, the spread of communicable diseases is highly significant and distinctive^7^. Participants in the Arbaeen pilgrimage may be exposed to the risks of communicable diseases such as water and foodborne illnesses, injuries and traumas, terrorism, and bioterrorism^8^.

In the Arbaeen, given the large and variable population and the fluctuating travel times of pilgrims, health authorities should be prepared to manage the extra pressure on the healthcare system^9^. Besides, ensuring the safety and health of participants in this event is one of the most important parameters in the successful management of mass gatherings^10^. Among the common challenges in the Arbaeen, issues such as lack of appropriate transportation systems, inadequate sanitary facilities, limited access to safe drinking water, communicable diseases, overcrowding, and shortages of medicine and medical equipment can be mentioned^11–13^.

The research gap identified is limited information and lack of studies conducted by researchers, there have been very few investigations into the challenges of providing healthcare services during the Arbaeen. Most studies in this regard have been quantitative and cross-sectional, usually examining formal structures, but have not analyzed informal processes, how groups coordinate, and the momentary decisions made during the event. On the other hand, through a qualitative study, more hidden challenges usually overlooked by the general public can be identified and a conceptual model can be derived to systematically explain the challenges. Identifying the problems and challenges of service delivery can significantly contribute to the safer organization of this global religious event in the future. Therefore, this study aimed to elucidate the perspectives of healthcare providers regarding the operational challenges of providing services to pilgrims during the Arbaeen in 2023.

## Methods

### Study design

This study employs a descriptive qualitative approach through the use of content analysis. This study explored the viewpoints of healthcare providers, as those with experience and expertise in healthcare services can assess current challenges and obstacles from various perspectives. Additionally, their daily involvement in organizational issues offers valuable insights into structural problems, human resources, and administrative processes. Their insights into existing challenges can contribute to developing effective solutions to enhance healthcare services.

### Research setting

**Fig. 1:**
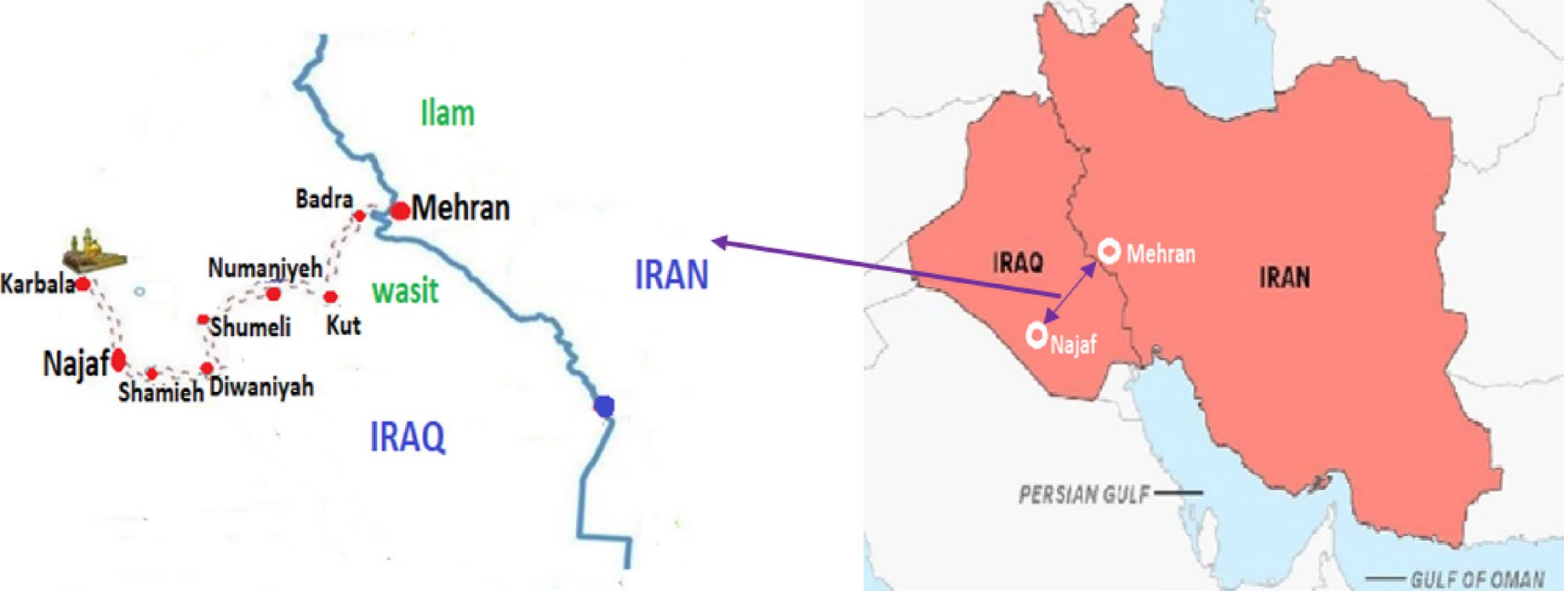
Pilgrims' walking route from the Mehran border to the cities of Najaf and Karbala.

The research setting in this study included mobile clinics providing Iranian-affiliated health services located in Wasit Province, Iraq. Wasit Province is one of the provinces of Iraq located in the east of the country and on the border of the country with Iran. The Mehran border in Ilam Province in Iran is considered one of the six land borders for Arbaeen pilgrims to cross every year. Mehran is the busiest border for Arbaeen pilgrims, and about 60 percent of pilgrims travel through the Mehran border. The distance from the Mehran border to Najaf is 303 km, and after crossing the Mehran border, pilgrims must pass through the cities of Badra, Kut, Numaniyah, Shumali, and Diwaniyah to reach Najaf^14^. Fig 1.

### Sampling procedure and participants

This study was conducted through in-depth interviews based on the experiences of healthcare providers in the Arbaeen and mobile clinics, including general practitioners, emergency medical technicians, nurses, and nurse assistants.

Participants were selected from healthcare providers in mobile clinics affiliated with relevant organizations such as the Red Crescent, the Hajj and Pilgrimage Organization, and the Medical Society of Universities of Medical Sciences using purposive and snowball sampling methods to maximize diversity. Purposive sampling is a commonly utilized technique in qualitative research aimed at identifying and selecting cases that provide rich information, thereby optimizing the use of limited resources. This method involves choosing individuals or groups who possess relevant experience and knowledge about the phenomenon being investigated. Furthermore, these participants must be accessible, willing to engage, and capable of articulating their experiences and insights in a thoughtful and expressive way^15^. The inclusion criteria included sufficient experience in implementation (a minimum of three years in dealing with this event), the ability to establish communication, and willingness to participate in interviews.

### Data collection

In this study, face-to-face interview by a professional interviewer were conducted until saturation. Data were collected using an audio recording device with written permission from the participants. Open-ended questions were designed by experienced policymakers and healthcare providers to generate data. The duration of the interviews ranged from 60 to 75 min, depending on the participants’ tolerance, level of information, and willingness.

Semi-structured interviews were conducted with participants after obtaining informed consent. To ensure confidentiality, all audio recordings and transcripts were stored on a password-protected personal computer, accessible only to the principal investigator. Identifiable information was removed, and participants were assigned unique confidential codes (e.g., N-1, N-2 for Nurses; GP-1, GP-2 for General practitioners). Physical notes were kept in a locked cabinet, and digital files were encrypted.

The interviews began with the following general questions: “How is your organization’s program for providing healthcare services to pilgrims during the Arbaeen?”, “ what are the problems and challenges in providing health services in mobile centers in providing services to patients,” and “ What measures have been taken by the authorities to address the challenges?” Following these questions, exploratory questions were gradually used to clarify concepts and gain deeper insights during the interview process. For example, “Please provide further explanation”, “What do you mean by that?” and “Why?” were used. (Appendix 1).

Data collection lasted from August 18, 2023 to September 22, 2023 during the Arbaeen pilgrimage, and the entire research was conducted between July 20, 2023 and January 2024.

### Data analysis

The data were analyzed using inductive content analysis. Initially, the primary researcher transcribed the interviews into written text. The digital files were listened to multiple times, and the written text was read several times. Then, meaningful units were identified based on the research objectives and questions. These meaningful units were sets of words and sentences that were conceptually related to each other and placed together. When the meaningful units reached a level of abstraction and conceptualization, coding was performed. Initially, key points were extracted as open codes. These codes were then grouped into broader categories based on their similarities and differences. In other words, the data were reduced to describe the phenomenon and achieve a better understanding, and this abstraction process continued until concept extraction^16^.

### Rigor

The Guba and Lincoln method was utilized to assess the data’s rigor and trustworthiness (credibility, dependability, confirmability, and transferability).

The credibility of the findings was ensured through prolonged researchers involvement across various study stages. This included reviewing interview content, multiple readings of transcripts, participant feedback on transcripts, and revisions based on that feedback. Also sampling with a maximum variation of age, gender, marital status, type of employment, job, education, place of work, years of work experience and techniques such as active listening, extended engagement with the data, immersive data analysis, and triangulation of data sources and investigators were employed.

Confirmability was achieved by sharing coded interview data samples with some participants to gather feedback on the analysis accuracy and by having an expert in qualitative methods review the interviews.

To transferability, the researchers offered thorough information about the research setting, participant experiences, data analysis, and study results, allowing readers to determine how applicable the findings might be to other contexts.

Recording all procedures taken to conduct the study and conducting all interviews under similar conditions improved the dependability of the data.

## Results

According to the data presented in Table 1, a total of 19 healthcare providers participated in the interviews. Among them, 8 were employed in clinics affiliated with the Ministry of Health and Medical Education (MoHME), 6 were working in clinics affiliated with the Hajj and Pilgrimage Organization, and 5 were associated with clinics affiliated with the Iranian Red Crescent Society. The majority of the participants were male (n = 12; 63.2%), married (n = 11; 57.9%), had permanent employment contracts (n = 11; 57.9%), and held MD’s and bachelor’s degrees (n = 7; 36.8%). (Detailed information about the participants is provided in Appendix 2).

**Table 1:** Demographic variables of participants in the study.

Variable	Number (Percentage) Mean ± SD
Age (years)	35.9 ± 5.3
Experience (years)	14.21 ± 5
Affiliation of clinics	
MoHME	8(42.1)
Hajj and Pilgrimage Organization	6(31.5)
Iranian Red Crescent Society	5(26.4)
Gender	
Female	7(36.8)
Male	12(63.2)
Marital status	
Married	11(57.9)
Single	8(42.1)
Type of employment	
Permanent	11(57.9)
Temporary	8(42.1)
Education	
Associate degree	3(15.8)
Bachelor	7(36.8)
Master	2(10.6)
MD	7(36.8)
Job	
Nurse	5(26.3)
Emergency medical technician	4(21)
General practitioner	7(36.8)
Nurse assistant	3(15.9)

The findings regarding the challenges faced by mobile healthcare service providers during the Arbaeen were identified across three main categories (infrastructure, diseases and treatment and pilgrims) and nine subcategories (human resources, physical space, capital medical equipment, medications and medical supplies, communicable diseases, non-communicable diseases, inadequate understanding of potential risks by pilgrims, physical disabilities of some pilgrims, medication-related issues) (Table 2).

**Table 2:** Challenges of providing healthcare services in mobile clinics during the Arbaeen.

Theme	Sub-theme	Primary code
Infrastructure	Human resources	Shortage of specialized staff
		Lack of motivation among healthcare workers to provide services in non-holy sites
		High turnover of healthcare staff
		Mismatch in the skill mix of service providers
		lack of proficiency in Arabic language among healthcare workers
	Physical space	Lack of physical space for staff rest
		Absence of a cold room
		Insufficient physical space for providing care services
	Capital medical equipment	Shortage of ambulances
		Shortage of autoclaves
	Medications and medical supplies	Shortage of anti-diarrheal and anti-emetic medications
		Shortage of masks
		Shortage of hand sanitizers
Diseases and treatment	Communicable diseases	Respiratory diseases among pilgrims and staff
		Gastrointestinal diseases among pilgrims and staff
	Non-communicable diseases	Heat exhaustion and skin diseases
		Musculoskeletal injuries
Pilgrims	Inadequate understanding of potential risks by pilgrims	Ineffectiveness of training programs:
		Beliefs and attitudes
	Physical disabilities of some pilgrims	Disabled individuals
		Children
		Pregnant women
		Patients with complex conditions
	Medication-related issues	Absence of necessary medications
		Self-medication practices

### Challenge related to infrastructure

#### Human resources

The challenges related to human resources were one of the key topics addressed in this study. These challenges were identified through codes such as shortage of specialized staff, lack of motivation among healthcare workers to provide services in non-holy sites, high turnover of healthcare staff, mismatch in the skill combination of service providers, and lack of proficiency in Arabic language among healthcare workers. In this regard, some of the interviewees expressed:

“At times, there were so many patients that we didn’t know which one to attend to. We had a high number of patients and a shortage of staff.” (Participant 2, Nurse)

“They should prioritize securing specialized staff before starting the ceremonies. This way, both the pilgrims and the volunteers who serve them would not be disadvantaged.” (Participant 5, GP)

“Because this center is far from Najaf and Karbala, very few people are willing to serve here. Most individuals prefer to be in centers close to the holy shrines.” (Participant 17, Nurse assistant)

“After a while in the center, the staff no longer had the desire to stay, and this made us constantly think about finding replacements. The staff would leave the center without a replacement.” (Participant 6, Nurse)

“We had nursing staff, but we had a shortage of nursing assistants and support staff. The nurses themselves had to perform all the tasks, and the workload became overwhelming.” (Participant 4, Nurse)

“In most cases, due to the shortage of nursing assistants, other staff members, such as Nurses, end up performing nursing assistant duties.” (Participant 12, GP)

#### Physical space

The challenge of inadequate physical space was identified through three codes: lack of space for healthcare staff rest, lack of space for service provision, and absence of a cold room. In this regard, some participants expressed:

“Some mobile centers do not have enough capacity to accommodate patients, and we are forced to provide services to patients outside the center.” (Participant 16, GP)

“We did not have sufficient and suitable space for rest. Sometimes, after working for 48 h, we wanted to take a break, but unfortunately, there was no suitable space for rest.” (Participant 10, GP)

“Due to the lack of physical space for service provision, we had to use the staff rest area for patients, and we did not have a place for ourselves to rest.” (Participant 6, Nurse)

“We did not have a cold room for patients who had a high body temperature. As a result, patients would stay in the centers for a long time until they felt better. If we had a cold room, patients would recover faster, and it would reduce the gathering of patients who were mostly affected by the heat.” (Participant 10, GP)

#### Capital medical equipment

The challenge of inadequate capital medical equipment was identified through two codes: Shortage of ambulance and Shortage of autoclaves. In this regard, some participants expressed:

“Sometimes we need to transfer a patient quickly to a more equipped center, but we don’t have enough ambulances.” (Participant 3, GP)

“Perhaps we don’t have a shortage of ambulances in terms of numbers, but when there is such congestion of people on the roads, a greater number of ambulances should be anticipated. When an ambulance takes four hours to cover a route that normally takes an hour, it indicates a shortage of ambulances.” (Participant 7, GP)

“In all centers, there is no autoclave. It might be available in centers affiliated with medical universities, but not in our center.” (Participant 2, Nurse)

“One cannot feel confident about the performance of these autoclaves. It seems like they have brought old and forgotten autoclaves here.” (Participant 19, Nurse assistant)

#### Medications and medical supplies

“Shortage of medications and medical supplies” was identified through three primary codes; “ shortage of anti-diarrheal and anti-emetic medications”, “ shortage of masks,” and “ shortage of hand sanitizers.” In this regard, some participants expressed:

“Considering that the majority of our patients had gastrointestinal issues, there were times when we experienced a shortage of anti-diarrheal and anti- emetic medications.” (Participant 9, EMT)

“In the beginning, we had shortages of hand sanitizers and masks, but this problem was less prevalent last year.” (Participant 5, GP)

#### Diseases and treatment

This category has been identified with three subcategories: communicable diseases, non-communicable diseases, and shortages of medication and medical supplies.

#### Communicable diseases

This subcategory has two primary codes: respiratory diseases among pilgrims and staff and gastrointestinal diseases among pilgrims and staff. Some participants expressed the following:

“Unfortunately, due to the lack of adherence to hygiene principles, we are witnessing an increase in respiratory and gastrointestinal diseases. If pilgrims would take some precautions, many of these diseases could be prevented.” (Participant 14, Nurse)

#### Non-communicable diseases

This subcategory consists of two primary codes: heat-related illnesses and skin diseases, and musculoskeletal injuries. Some participants expressed the following:

“The heat intensity is so high that even young pilgrims are affected by heat exhaustion. Those who do not use hats and masks are particularly prone to severe sunburn.” (Participant 11, EMT)

“Swollen feet, muscle cramps, and knee pain are among the main complaints of pilgrims, and there are not many solutions available for them. Many of the palliative treatments are added to these statistics alongside minor accidents.” (Participant 16, GP)

#### Challenges related to pilgrims

This category consists of three subcategories:

#### Inadequate understanding of potential hazards among pilgrims

This subcategory consists of two primary codes: Ineffectiveness of training programs and beliefs and attitudes. Some participants expressed the following:

“No matter what we teach them, it has no effect. Sometimes it even backfires, and individuals invite us to adhere to their false beliefs.” (Participant 15, EMT)

“In my opinion, education has no benefit for these individuals (strongly religious ones), and clerics should take responsibility for these teachings. However, one of our problems is that some of these clerics have contributed to the creation of these beliefs and convictions among people!” (Participant 8, Nurse)

“No matter what education we provide to pilgrims, they respond by saying it is impossible for them to get sick. They believe they are the guests of the Lord, and the Lord will protect them!” (Participant 12, GP)

“Unfortunately, some pilgrims, despite knowing and seeing that the distributed drinks are prepared without adhering to hygiene principles, still consume them due to their beliefs.” (Participant 4, Nurse)

“Some individuals, due to their beliefs, walk the route without using hats and masks to protect themselves from direct sunlight and heat, and as a result, they often experience problems.” (Participant 14, Nurse)

#### Physical disabilities of some pilgrims

Individuals with disabilities, children, pregnant women, and critically ill patients.

The presence of individuals without any limitations in the Arbaeen, unlike the Hajj pilgrimage which imposes age restrictions and considers specific physical abilities for participation, poses challenges for healthcare providers. In this regard, some participants expressed the following:

“When individuals of all age groups participate in the Arbaeen, healthcare providers are expected to have comprehensive medical knowledge. However, due to the existing shortage of personnel, this becomes practically impossible. It would be beneficial if age restrictions were also implemented for this pilgrimage.” (Participant 2, Nurse)

“Some pilgrims do not have the physical ability to undertake this journey. For example, pregnant women not only risk harm to themselves but also potential harm to their unborn child. Similarly, pilgrims with cancer embark on this journey with hopes of healing. All these cases create numerous challenges for us.” (Participant 1, GP)

“Some pilgrims have very limited physical abilities or disabilities, yet they choose to embark on this path. Many of them struggle along the way, lacking the energy to continue or the means to return. These individuals pose significant challenges for us.” (Participant 8, Nurse)

#### Issues related to the medications used by pilgrims

The absence of medications and Self-medication practices were identified as other codes by the interviewees. Some pilgrims who had underlying or difficult-to-treat conditions did not have their necessary medications with them or an adequate supply. In this regard, some participants acknowledged the following:

“The biggest problem we face is when a patient with multiple underlying conditions arrives but has not brought their medications or has lost them along the way. These cases create the most significant challenges for us.” (Participant 11, EMT)

“Our main issue with pilgrims is that, in addition to not bringing their medications, they are also unaware of the names of their medications. It becomes quite confusing for us because most of them have various medical conditions, and we don’t want to worsen their situation by providing them with the wrong medication.” (Participant 18, Nurse assistant)

“Some pilgrims, without informing the healthcare staff, would use chemical medications, traditional remedies, and, in some cases, even illicit drugs for pain relief, leading to complications in their ongoing treatment” (Participant 7, GP)

“We have encountered numerous instances where a person experiencing foot or knee pain did not have their own medication but took a capsule from another pilgrim. Subsequently, they experienced stomach discomfort, and when asked about the name of the medication, they would respond with uncertainty, saying, “I don’t know! Only God knows what was in that capsule!” (Participant 11, EMT)

## Discussion

This study aimed to examine the challenges faced by mobile healthcare providers in delivering care services to pilgrims during the Arbaeen from the perspective of service providers. The main findings of this study revealed challenges related to human resources, physical space, medical equipment, communicable and non-communicable diseases, shortage of medications and medical supplies, inadequate understanding of potential hazards by pilgrims, physical limitations of some pilgrims, medication-related issues, and communication.

The first challenge identified in this study was a set of challenges related to human resources, including a shortage of specialized staff, a lack of motivation among employees to provide services in centers far from the holy shrines, a high turnover of healthcare staff, a mismatch in the skill combination of service providers, and insufficient proficiency in the Arabic language.

Peirovi et al. highlighted the inadequacy of both quantitative and qualitative trained personnel as one of the challenges related to health in the Arbaeen^17^. Delgado refers to the significance of a cohesive, multidisciplinary, and motivated team with common goals and the ability to adapt to changing environments as a crucial aspect for any complex project^18^. Similarly, McLoughlin et al. discuss staffing shortages in rural hospitals and emphasize the necessity of precise planning and accurate estimation of human resources to ensure the provision of satisfactory healthcare services^19^. In a similar study, the ratio of paramedics to physicians in mobile clinics was found to be 1.4 to 1, which is close to the ratio in Iraq (1.8 to 1)^20^. Considering the annual occurrence of the Arbaeen, which lasts for approximately 20 days, predicting and estimating the required human resources should not pose a significant challenge for the responsible authorities, and this challenge can be overcome through meticulous planning. Factors such as inadequate planning and forecasting of manpower needs before the event, lack of accurate information on the number of pilgrims and their health needs, lack of financial resources, lack of staff motivation, and language and cultural issues seem to be contributing factors. To address these challenges, we can improve planning and forecasting of needs, attract and retain skilled workers through financial and welfare incentives, hold training courses to increase specialized skills and Arabic language proficiency, and create effective communication between service providers and health managers.

The second identified challenge in the present study was the lack of suitable physical space. Within the context of insufficient physical space for healthcare staff’s rest, the absence of a cold room, and the shortage of physical space for providing medical and care services were identified. It is evident that having appropriate space for service recipients and healthcare personnel is a fundamental element for improving service delivery to pilgrims. Similarly, Rosouli et al. refer to the inadequacy of facilities related to medical centers and rest areas as one of the challenges in service provision during the Arbaeen^21^. Lack of attention to infrastructure needs during the event Despite past experience, officials may not have access to the financial, administrative, and logistical resources needed to provide a suitable space or may not pay enough attention to addressing these problems. Also, lack of coordination between different organizations and lack of a comprehensive plan for managing difficult and emergency situations during the event could lead to the continuation of these challenges.

The next identified challenge in this study relates to the shortage of capital medical equipment, specifically the lack of ambulances and autoclaves. Another study showed that the absence of sufficient pre-hospital medical services and ambulances is a risk factor for the population in mass gatherings^22^. Lami et al. demonstrated that only 40% of service providers had access to ambulances for transporting patients to appropriate hospitals or healthcare centers^23^. Soltani also suggests that planning for the rapid transfer of injured patients and pilgrims, as well as providing on-site triage at the scene of the incident, should be considered to improve service delivery^24^. This challenge can be partly attributed to infrastructural weaknesses and partly to road congestion in crowded gatherings.

The autoclave was identified as another shortage mentioned by the interviewees, similar to the findings of Lami et al.^20^. Considering that the unavailability of sterilization equipment can increase the risk of infection, responsible authorities need to ensure the availability of such equipment in all clinics affiliated and non-affiliated with the MoHME.

It appears that there is a lack of planning and allocation of appropriate resources by health and medical authorities. Despite the annual holding of this event, the lack of a detailed assessment and pathology of past experiences has led to a failure to identify and address infrastructure and logistical problems. Also, the necessary funding for the purchase and maintenance of equipment on an ongoing basis may not be a priority for health managers, leading to a lack of adequate preparation to respond to the health needs of pilgrims.

Based on the current study, the shortage of medications, serums, and medical supplies, including masks and hand wrap, was another challenge faced by mobile care centers during the Arbaeen. Lami et al. demonstrated that only half of the temporary clinics located in Mawakeeb (temporary settlements) had medications for blood pressure and diabetes. Additionally, almost all centers had essential sanitary supplies for infection control, except for hand wrap, which were available in only about 60% of the centers^20^.

It seems that the shortage of medication and medical supplies in mobile clinics during the Arbaeen pilgrimage is due to several reasons. Among them, the lack of adequate planning and forecasting by officials leads to a shortage of equipment and raw materials during peak times of pilgrim visits. Additionally, considering the steadily increasing number of pilgrims and the high volume of medical services during this period, the existing capacity is insufficient. Other factors include resource supply limitations, logistical issues in distribution, disorganization in the distribution and storage of items, and coordination problems among the responsible entities in providing health services.

The next identified challenge pertains to the domain of diseases and healthcare, specifically communicable and non-communicable diseases, as well as the shortage of medication and medical supplies. According to the existing literature, symptoms of acute respiratory illnesses, such as influenza, were the most common complaints among pilgrims visiting mobile clinics in mass gatherings during Arbaeen^2,20,25,26^. Considering the high prevalence of respiratory diseases among pilgrims during the Arbaeen, one of the main health risks for pilgrims is related to upper respiratory tract infections. Using masks, adherence to hygiene principles by the public, and practical education can help prevent such diseases. Studies also indicate a high prevalence of gastrointestinal complaints among visitors to mobile healthcare centers during the Arbaeen ^20,27,28^. Monitoring the preparation and distribution of food and beverages provided along the pedestrian route to Najaf and Karbala can help prevent the spread of gastrointestinal diseases. Some studies have reported contamination in samples of consumed water^29^ and fecal samples of gastrointestinal patients^30^.

Non-communicable diseases such as heat exhaustion, sunburn-related skin diseases, and skeletal and muscular traumatic injuries mainly resulting from road accidents were among the cases that led to an increased number of visits to mobile healthcare centers. In this regard, a study conducted in 2014 to evaluate surgeries reported in hospitals and temporary healthcare centers during the Arbaeen showed that road accidents and violence were the main causes of mortality in this event^22^. Another study highlighted the absence of safe public transportation, inadequate roads considering weather conditions, parking issues, and the limited entry of private vehicles into Iraq as significant challenges in this domain^21^. Heat exhaustion and skin diseases due to sunburn were also addressed as important considerations. Rasouli et al. mentioned heat exhaustion as one of the problems faced by pilgrims, especially during the hot season^21^.

The spread of communicable and non-communicable diseases among pilgrims during the Arbaeen pilgrimage can lead to disruptions in the provision of effective services to patients for various reasons. One of the main factors is the sudden increase in the number of patients, which puts pressure on the capacity of mobile clinics; as a result, medical personnel may not be able to provide appropriate and timely care to all patients. Also, the presence of communicable diseases such as influenza in large and close-knit populations can have a wider spread and affect the number of visits to medical centers. On the other hand, non-communicable diseases such as heatstroke and sunburn increase during the long pilgrimage, which also puts pressure on the demand for medical services. These conditions ultimately lead to insufficient time and resources for medical personnel, as well as a shortage of medicines and equipment, which reduces the efficiency of medical teams and can lead to health crises.

The third identified challenge in this study was related to the recipients of services. It encompassed several aspects, including insufficient understanding of health hazards by pilgrims, physical incapacity in some pilgrims, and the lack of specific and necessary medications related to underlying medical conditions among pilgrims.

Understanding health-threatening hazards is a priority in reducing diseases, and it requires a preventive approach based on public education. The Sustainable Development Goals (SDGs) emphasize the role of education in increasing awareness of hazards and reducing vulnerability to potential risks^21^. The results of a study on beliefs and infection control methods among pilgrims showed that the majority of participants had a limited understanding of respiratory infections and the need for vaccination during the pilgrimage^31^. During the Hajj pilgrimage, for example, millions of Muslims from around the world gather in Saudi Arabia each year, creating a high-risk environment for the spread of VPDs. In 2019, the Saudi Ministry of Health reported that over 1.8 million doses of vaccines were administered to pilgrims, protecting them against diseases such as meningococcal meningitis, yellow fever, and seasonal influenza^32^. Islamic teachings state that individuals are obligated to protect their health and lives in any place or situation, even in sacred sites, and to avoid hazards. Since Arbaeen is a religious gathering, religious leaders have an important role in the implementation of the rituals and can have an influential impact on the beliefs and understanding of pilgrims regarding pilgrimage hazards.

One of the challenges identified in this study was the physical inability of individuals to participate in the Arbaeen rituals due to old age and multiple underlying diseases. Additionally, the lack of certain specific medications that patients take, such as cardiovascular and cancer medications, further complicated the provision of services to these patients. In some cases, this led to delays in providing care and, tragically, resulted in the deaths of patients. Hantoosh et al. identified cardiovascular diseases as the main cause of mortality during the 2014 Arbaeen^32^. Furthermore, cardiovascular diseases were identified as the leading cause of mortality among Iranian patients hospitalized in Iraqi hospitals^33^.

Another issue prevalent among pilgrims is the practice of self-medication, which aligns with the findings of Karimpoorian’s study^11^. Furthermore, a study evaluating the knowledge, attitudes, and practices of Australian pilgrims revealed that the research sample had a poor understanding of antibiotic use and engaged in self-administration of these medications^34^.

It seems that pilgrims’ lack of awareness of health risks may lead them to adopt risky behaviors and neglect health tips, which will lead to more diseases and complications. This will significantly increase the volume of visits to clinics and, in turn, put more pressure on medical personnel. The physical incapacity of pilgrims, especially in the elderly or those with chronic diseases, can reduce their strength and capacity to walk and endure difficult conditions during the ceremony, and as a result, they need more specialized and urgent care. This can also lead to a decrease in the quality of services provided to these patients. Not having essential medicines with them when traveling and self-medication practices can have serious consequences, especially for people with certain diseases. In the event of complications resulting from lack of access to medication, the need for emergency services increases, which can undermine the lack of capacity and treatment facilities available in mobile clinics and even lead to critical and emergency cases.

Although interviewees did not directly mention a lack of cooperation between the countries involved, similar experiences (such as the Hajj pilgrimage or large international gatherings) suggest that such cooperation can reduce challenges in securing human and financial resources. It is suggested that in the future, more formal mechanisms between host and sending countries be designed to improve health services.

### Limitations and strengths of the study

This qualitative research is one of the few studies conducted on the experiences of Arbaeen, so it provides rich information in this field. Since the results of this study were collected from semi-structured interviews, they are considered subjective. Therefore, it is suggested that future studies should objectively transform and analyze subjective concepts by creating quantitative tools to examine the challenges and measure them. The present study can be used as a basis for this purpose. This research was based on the perspectives of interviewees (mainly healthcare professionals). Therefore, issues such as international cooperation, which require high-level policy-making involvement, are not reflected in the data. Given the importance of coordination between countries in managing major events, it is recommended that studies be conducted focusing on cooperation mechanisms between Iran, Iraq, and other pilgrim-sending countries. These studies could include examining joint funding models, deploying specialized forces, and establishing unified health protocols.

## Conclusion

Considering the identified challenges associated with this massive human event that takes place annually with a growing population, the need for planning at three levels is felt for better health and medical services for the pilgrims.

Before departure; the necessity of maximum vaccination of individuals to prevent diseases and their transmission, providing medical facilities for the pilgrim population, estimation of the required human resources in terms of numbers and specialized skills in healthcare facilities, distribution of personal hygiene items, especially masks, among individuals, informing pilgrims about personal hygiene practices, coordination between healthcare organizations involved in organizing the event between the two countries, recording the medical summaries of pilgrims and recommending the possession of specific medications.

During the event; population control through coordination among event organizers, coordination between departments for the distribution of drugs and healthcare facilities, negotiation with the destination country to establish accommodation centers, and proper waste collection, planning, and coordination between service units and foreign organizations such as insurance companies.

After the event; arrangements for the gradual and non-congested entry of pilgrims, control of communicable diseases at the country’s entry points and quarantine facilities for infected individuals, and planning for the rapid transfer of critically ill patients to domestic healthcare centers.

## Author contributions 

SST and MA conceived the study. MA supervised all evaluation phases and revised the manuscript. ‎SST and MA conducted primary data analysis. All authors read and approved the final ‎version of the manuscript.

## Funding 

This study has been approved and funded by Mashhad University of Medical Sciences.

## Supplementary Information

Below is the link to the electronic supplementary material.

Supplementary Material 1

Supplementary Material 2

## Data Availability

All data generated or analyzed during this study are included in this published article.

## Abbreviations

MUMS: Mashhad University of Medical Sciences
WHO: World Health Organization
MOHME: Ministry of Health and Medical Education
GP: General practitioner
EMT: Emergency medical technician
MD: Medicine doctor
