# Supplementary material for: A qualitative study on operational challenges of Iranian affiliated mobile health clinics in Iraq during Arbaeen
Source: Sci Rep. 2025 Nov 6;15:38935. doi: 10.1038/s41598-025-22820-7 (PMC12592363; doi:10.1038/s41598-025-22820-7)
Supplement: Supplementary file 1 — Supplementary Material 1 [file 41598_2025_22820_MOESM1_ESM.pdf]

Appendix 1: Interview Guide (English Translation)

Name: ___________________________________

Surname: _________________________________

Age: marriage:

Experience:

Date of Interview: _________________________

Name of Health Center: _____________________

Interviewer's Name: _________________________

Introduction: Thank you for agreeing to participate in this interview. Your insights and explanations are valuable and important. This interview is structured as a general conversation and is completely informal. I am conducting a study to understand the challenges of providing healthcare services in Iranian-affiliated mobile clinics based in Iraq during the Arbaeen ceremony. I am very interested in learning your thoughts and explanations. There are no right or wrong answers. I would like to hear both your positive and negative experiences. The findings from this interview, along with those from your colleagues, will help create a foundation for improving quality services among healthcare centers. This interview will take approximately ... minutes. I would like to record your voice for accuracy. Please note that this interview is completely confidential, and you may stop the interview at any time if you wish. I kindly ask you to complete and sign the informed consent form before we proceed. We aim to understand your perspective on the Challenges of Providing Healthcare Services in Iranian-affiliated mobile clinics, so please note that I may occasionally change the topic or move past some questions. However, feel free to add anything you find important.

Consent Statement: I, _________________________________, hereby declare that I fully understand the objectives of this study and voluntarily consent to participate. I commit to providing honest responses to the questions.

Signature: ___________________________________

Interview Questions:

Warm-up / Opening Question:

I would like to know how many times you have participated in Arbaeen as a healthcare provider in mobile clinics? Can you tell us about your work experience?

Main Questions:

How is your organization's plan for providing health services to Arbaeen pilgrims? Is it fully prepared to provide services?

In your opinion, what are the problems and challenges in providing health services in mobile centers in providing services to patients?

What measures have been taken by the authorities to address the challenges? Were the problems addressed quickly? Are these challenges expected to be resolved next year?

In your opinion, what risks does the Arbaeen walking ceremony pose to people's health and how does it threaten people's health?

What measures are foreseen to monitor and control these risks and threats?

Final question:

Please state if there is an important issue in your opinion that has not been addressed.
