# Supplementary material for: A qualitative study on operational challenges of Iranian affiliated mobile health clinics in Iraq during Arbaeen
Source: Sci Rep. 2025 Nov 6;15:38935. doi: 10.1038/s41598-025-22820-7 (PMC12592363; doi:10.1038/s41598-025-22820-7)
Supplement: Supplementary file 2 — Supplementary Material 2 [file 41598_2025_22820_MOESM2_ESM.pdf]

Participant number	Age	Gender	Marital status	Type of employment	Education	Job	Work experience	Affiliation of clinics
P1	40	Male	Married	Permanent	MD	GP	18	MoHME
P2	27	Female	Married	Temporary	Bachelor	Nurse	7	Hajj and Pilgrimage Organization
P3	32	Male	Single	Temporary	MD	GP	12	Iranian Red Crescent Society
P4	30	Female	Married	Temporary	Master	Nurse	9	MoHME
P5	42	Male	Married	Permanent	MD	GP	20	Iranian Red Crescent Society
P6	42	Female	Married	Permanent	Master	Nurse	21	Hajj and Pilgrimage Organization
P7	41	Male	Single	Permanent	MD	GP	20	Iranian Red Crescent Society
P8	30	Female	Married	Temporary	Bachelor	Nurse	8	MoHME
P9	29	Male	Single	Temporary	Bachelor	EMT	8	MoHME
P10	35	Female	Married	Permanent	MD	GP	15	Iranian Red Crescent Society
P11	33	Female	Married	Permanent	Bachelor	EMT	14	MoHME
P12	38	Male	Married	Permanent	MD	GP	17	Iranian Red Crescent Society
P13	44	Male	Married	Permanent	Bachelor	EMT	22	Hajj and Pilgrimage Organization
P14	40	Female	Married	Permanent	Bachelor	Nurse	18	Hajj and Pilgrimage Organization
P15	28	Male	Single	Temporary	Bachelor	EMT	6	MoHME
P16	36	Male	Single	Permanent	MD	GP	12	Hajj and Pilgrimage Organization
P17	38	Male	Single	Temporary	Associate degree	Nurse assistant	15	MoHME
P18	40	Male	Married	Permanent	Associate degree	Nurse assistant	16	Hajj and Pilgrimage Organization
P19	38	Male	Single	Temporary	Associate degree	Nurse assistant	12	MoHME
